# Tunneling Nanotubes Provide a Unique Conduit for Intercellular Transfer of Cellular Contents in Human Malignant Pleural Mesothelioma

**DOI:** 10.1371/journal.pone.0033093

**Published:** 2012-03-09

**Authors:** Emil Lou, Sho Fujisawa, Alexei Morozov, Afsar Barlas, Yevgeniy Romin, Yildirim Dogan, Sepideh Gholami, André L. Moreira, Katia Manova-Todorova, Malcolm A. S. Moore

**Affiliations:** 1 Department of Medicine, Memorial Sloan-Kettering Cancer Center, New York, New York, United States of America; 2 Moore Laboratory, Department of Cell Biology, Sloan-Kettering Institute, Memorial Sloan-Kettering Cancer Center, New York, New York, United States of America; 3 Molecular Cytology, Memorial Sloan-Kettering Cancer Center, New York, New York, United States of America; 4 Department of Surgery, Memorial Sloan-Kettering Cancer Center, New York, New York, United States of America; 5 Department of Pathology, Memorial Sloan-Kettering Cancer Center, New York, New York, United States of America; National Taiwan University Hospital, Taiwan

## Abstract

Tunneling nanotubes are long, non-adherent F-actin-based cytoplasmic extensions which connect proximal or distant cells and facilitate intercellular transfer. The identification of nanotubes has been limited to cell lines, and their role in cancer remains unclear. We detected tunneling nanotubes in mesothelioma cell lines and primary human mesothelioma cells. Using a low serum, hyperglycemic, acidic growth medium, we stimulated nanotube formation and bidirectional transfer of vesicles, proteins, and mitochondria between cells. Notably, nanotubes developed between malignant cells or between normal mesothelial cells, but not between malignant and normal cells. Immunofluorescent staining revealed their actin-based assembly and structure. Metformin and an mTor inhibitor, Everolimus, effectively suppressed nanotube formation. Confocal microscopy with 3-dimensional reconstructions of sectioned surgical specimens demonstrated for the first time the presence of nanotubes in human mesothelioma and lung adenocarcinoma tumor specimens. We provide the first evidence of tunneling nanotubes in human primary tumors and cancer cells and propose that these structures play an important role in cancer cell pathogenesis and invasion.

## Introduction

Intercellular communication is critical to cancer cell proliferation, coordination, and tumor invasion. The traditional paradigm of cancer cell communication is reliance on potentially inefficient diffusion of chemical signals between cells, specifically transfer of materials responsible for stimulating growth of neighboring cells and coordinating tumor invasion. Other potential avenues of cellular transfer between cancer cells have been explored, including gap junctions or their component proteins, connexins, and microvesicles or exosomes [1]–[3]. However, the precise mechanisms for communication between proximal and distant cancer cells remain to be identified.

Tunneling nanotubes (TnTs) are fine, long, non-adherent, actin-based cytoplasmic extensions first described in PC12, a cell line of rat pheochromocytoma [4]. The authors demonstrated cell-to-cell spread of endosomes via these extensions, which they termed tunneling nanotubules to distinguish them from adherent actin-based cell extensions, such as lamellopodia, filopodia, and invadopodia. Characteristic morphologic features distinguishing TnTs from other actin-based structures are their small diameter, cell-to-cell cytoplasmic connections, and non-adherence to the substratum when cultivated *in vitro* [4]. Cells can form multiple TnTs, and some form as many as 75 TnTs [5].

Similar structures known as membrane nanotubes, cytonemes, or intercellular [6] or epithelial [7] bridges have been investigated as a means of intercellular transfer. The differences between tunneling and non-tunneling/membrane nanotubes as well as cytonemes have been discussed extensively elsewhere [8], [9]. TnTs are open-ended tubes whose walls consist of a lipid bilayer contiguous with the plasma membrane; the lumen establishes a direct connection between the cytoplasm of the connected cells. TnT formation is largely generated by actin-driven protrusions of the cytoplasmic membrane which extend to outlying cells and can transmit cellular cargo and signals. Prior studies on non-cancer cells have documented transmission of prions [10], [11], retroviruses [12], [13], apoptotic signals [14], and calcium signals [5], [15] between cells connected via TnTs. They have been noted to form either by one cell extending a tubular cytoplasmic connection to another cell located at some distance (in contrast with gap junctions, which connect cells in tight proximity) or alternatively between cells already in close proximity and which then move apart via usual mechanisms of cell motility, allowing for continuation of intercellular communication even as the cells move in different directions [16].

While the initial observation of TnTs was made in a cell line of rat pheochromocytoma cells (PC12) [4], much of the literature to date has focused on non-cancer cells, such as myeloid, dendritic, natural killer (NK), and T cells [5], [15], [17], as well as mesenchymal stromal cells [18]. A recent study documented a similar occurrence in primary human renal epithelial cells [19]. Examination of TnTs or similar structures in cancer has thus far been limited to cell lines of adrenal, prostate, colon, and gliomatous origin [4], [6], [20], [21]. To our knowledge, the potential formation and role of TnTs in solid tumors has not been previously explored, and little is yet known about the full role of TnTs for cells in general.

In our study, we demonstrated that TnTs form between mesothelioma cells at both close and distant proximity, and provide an alternative means for intercellular communication in cancer. We examined TnTs in cultures of mesothelioma cells from cell lines, primary pleural effusion specimens from patients, and solid tumors following surgery. Confocal microscopy was used to capture images of TnTs connecting fixed and live cells. We used time-lapse imaging to investigate transfer of proteins, mitochondria, and Golgi vesicles between proximal and distant cells through TnTs. We examined the reproducibility of stimulating TnT formation under various media conditions, and established conditions that would suppress this formation. We present the first documented evidence that TnTs form between primary mesothelioma and lung adenocarcinoma cells in culture, as well as in solid tumors resected from patients.

## Results and Discussion

### Observation and stimulation of TnTs in mesothelioma

**Figure 1:**
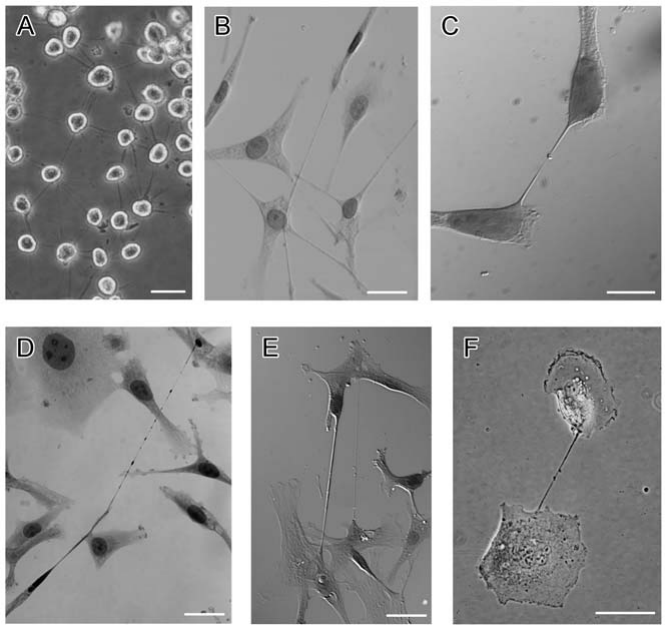
Tunneling nanotubes connect mesothelioma cells from cell lines and from human primary cancer cells. a) VAMT cells were trypsinized for over 60 minutes without disruption of TnTs. Multiple TnTs formed between cells. b) MSTO-211H cells were cultured to semi-confluence. Note that some TnTs pass over cells or other TnTs, demonstrating their characteristic non-adherence. c) Two MSTO-211H cells connected via a single TnT. d) MSTO-211H cells stained with pap stain. A long TnT passes over adherent cells and connects two cells at distant sites. e) Lung adenocarcinoma cells derived from a pleural effusion specimen from a human patient. One ultrathin and another, thicker TnT are noted, demonstrating the physical variability of TnTs in culture. f) Mesothelioma cells derived from a malignant pleural effusion from a human patient also demonstrate TnT formation with characteristic bulges representing transported cargo. Scale bars: all are 30 µm.

**Figure 2:**
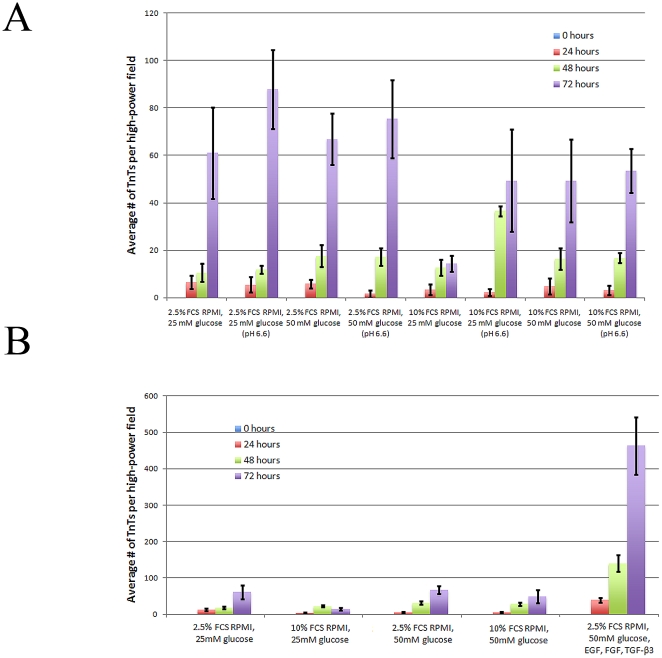
TnT formation can be stimulated by using a low-serum, hyperglycemic, acidic culture medium, or by inducing EMT. a) Live culture cells were examined using 20× objective lens on an inverted microscope; the number of TnTs in each field was counted, using 10 fields for each medium condition per experiment. b) Cells were counted as described above. Both experiments in this figure were performed in duplicate (n = 20).

TnTs can easily be overlooked as they span the three-dimensional plane *in vitro*, and are often out of the focal plane of adherent cells. Non-adherence is a key characteristic of TnTs and helps to distinguish them from more common adherent actin-based protrusions. We initially observed TnTs hovering above the substratum and connecting mesothelioma cells in mesothelioma cell lines (MSTO-211H, VAMT, H-Meso) cultured *in vitro*. The TnTs were highly resistant to trypsinization for more than 60 minutes, confirming previous observations [4] (**Figure 1A**). The characteristic findings of TnTs were consistent for cell lines as well as histologically-confirmed cancer cells from pleural effusions from patients with lung adenocarcinomas and mesothelioma (**Figures 1B–1F**). In all cases, TnTs formed spontaneously during *in vitro* growth in semi-confluent cultures, but were noted to be most prevalent in acidified hyperglycemic low-serum medium (pH 6.6, 50 mM glucose, 2.5% fetal calf serum) (**Figure 2A**), or in hyperglycemic, low-serum medium with cytokines added to stimulate epithelial-to-mesenchymal transition (EMT) (**Figure 2B**). TnTs formed within several hours of culturing cells once the cells began to adhere. They remained intact in semi-confluent cultures. Importantly, the number of cells per field was relatively constant over the first 72 hours of cell culture (**Supplemental Figure S1**). Notably, under normal acidity (pH 7.6), the highest cell count was observed for the cells in high serum (10% FCS) and normal glucose conditions, and with significantly less proliferation of cells grown in the low-serum (2.5% FCS), hyperglycemic medium which induced increased TnT formation. Thus we determined that an increase in numbers of TnTs was a reflection of an increase in *de novo* TnT formation, and not due to an increase in cell numbers from proliferation.

TnTs were distinguished by their non-adherence to the substratum, which was determined by observation that the nanotubes were thin and out of the standard focal plane. They were also noted to be somewhat mobile and oscillate upon movement of culture plates or following prolonged exposure to ambient light. Demonstration of the unique non-adherent nature of TnTs in the 3-dimensional plane *in vitro* is provided in the accompanying Supplementary Movies online (**Supplemental Movies S1 and S2**). TnTs were most apparent in cultures of low or moderate cell density, as cultures that were more confluent impaired detection of the nanotubes. We concluded that the structures we observed were TnTs due to characteristics such as non-adherence, resistance to trypsinization, and spontaneous formation that was further stimulated under specific metabolic conditions. A low-serum environment was most crucial to TnT formation. The combination of low serum with a hyperglycemic microenvironment was especially conducive. This finding is consistent with prior studies in which serum depletion elicited TnT formation in astrocytes under oxidative stress [22], and a study which suggested that hyperglycemia can induce actin-related morphological changes and cell extension in pericytes [23].

### TnT formation occurs during mesothelioma cell invasion *in vitro*

Low pH secondary to increased glycolysis and lactate secretion is a well-established property of proliferating and metastatic cancer cells. Furthermore, acidic pH has been reported to enhance the invasive potential of cancer cells [24]. Here we demonstrate that low pH in a hyperglycemic environment also stimulated TnT formation. To further demonstrate the potential association of TnT formation with cell invasion and proliferation, we performed scratch assays on adherent mesothelioma cells. MSTO-211H cells were cultured in the hyperglycemic, low serum, acidic medium for 72 hours to achieve confluence. A micropipette tip was used to gently scratch the cells and create a gap between two cell fronts, with addition of fresh medium. Time-lapse confocal microscopy was performed with images taken every 30 minutes for 24 hours. Images revealed regular formation of TnTs by proliferating and migrating cells that advanced to fill the gap (**Supplemental Movie S3**). This finding requires further evaluation to determine the role of TnTs developing between invasive cancer cells. Do TnTs stimulate increased proliferation and consequently increase invasive properties of these cells, or are invasive cells most likely to create TnTs to encourage signaling to nearby cells? Future studies will need to focus on this issue to eludicate the nature of TnT interactions between cancer cells, and whether or not these interactions are required for continued cell growth.

### Physical characteristics and composition of TnTs in mesothelioma

Identifying specific components of TnTs may lead to new approaches for therapeutic targeting of cell communication. Thus we performed immunofluorescent (IF) analysis of TnTs in mesothelioma.

**Figure 3:**
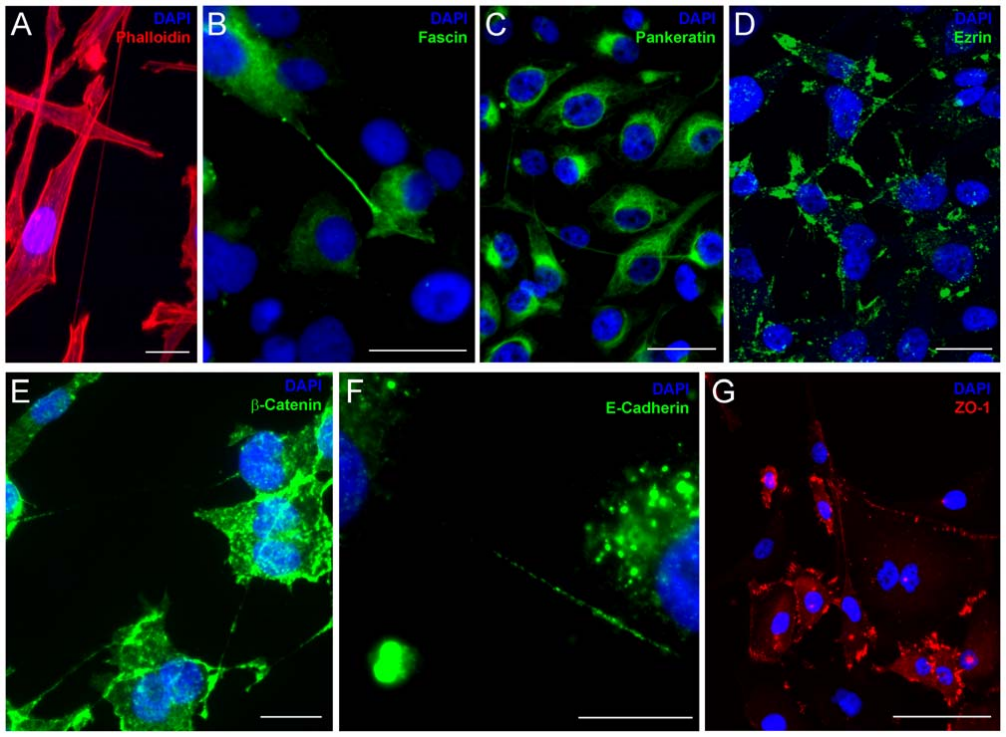
TnTs express proteins characteristic of actin-based extensions. Confocal imaging demonstrated immunofluorescence of specific protein components of MSTO-211H cells with stained TnTs. a) Actin is uniform across the entirety of the TnT, which passes over an adherent cell. b) Fascin is expressed intermittently and at the base of nanotubes c) Pankeratin localizes to the perinuclear region of the cells. d) Ezrin expression was most prominent at the base of TnTs, consistent with its role in organizing actin-based filaments. e) ß-catenin is minimally present within TnTs. f) E-cadherin staining of TnTs between cells. g) ZO-1 localizes to the cell membrane, including the point of contact of TnTs. Scale bars: a) 20 µm, b) 20 µm, c) 30 µm, d) 30 µm, e) 20 µm, f) 20 µm, g) 50 µm.

Phalloidin-rhodamine staining identified actin spanning the entire length of the TnTs (**Figure 3A**). We also identified fascin, a 55-kDa actin filament bundling protein regulated by protein kinase Cα; it facilitates dynamic cell extensions by cross-linking actin filaments [25]–[27]. It has been heavily implicated in initiation of cellular protrusions and subsequent distant metastasis [28]–[30]. We noted intermittent fascin expression over the length of TnTs, consistent with its known role in bundling actin in cell protrusions [31], [32]. Notably, fascin expression was most prominent at the base of nanotubes, specifically at the sites they extruded from the plasma membrane (**Figure 3B**). In many cases, staining was unipolar, and may indicate that fascin organizes and cross-links actin bundles at the origin of the TnTs which then extend to neighboring cells. Fascin has been associated with poorer prognosis of clinically advanced solid tumors [30], [33]–[35]; agents targeting fascin had been thought to demonstrate antitumor activity [36], but the initial reports have more recently come into doubt [37] and require further investigation. We postulate that fascin plays an important role in the formation of TnTs and may in part explain its role in aggressive tumor growth and proliferation.

Actin is also intimately connected with the ezrin-radixin-moesin complex of proteins and pancytokeratins, which assist in localization of actin bundles. In our study, IF staining demonstrated perinuclear localization of pancytokeratin in mesothelioma (**Figure 3C**). Ezrin (cytovillin) is a membrane-organizing phosphoprotein that mediates anchoring of actin microfilaments to cell membranes [38]–[40]. It has been implicated in organizing exosome formation and intercellular communication in mesothelioma, as well as organization of the base of actin filaments in microvilli and cellular extensions [1]. We found that expression of ezrin was highest at the site of extrusion of TnTs along the cytoplasmic membrane, supporting its role in the formation of these processes (**Figure 3D**). Cytosolic proteins intricately involved with cell adhesion and cell surface glycoproteins in adherens junctions are potentially involved in TnT formation as well. ß-catenin is a peripheral cytosolic protein normally found in many tissues, binding to the cytoplasmic tail domains of cell-adhesion cadherins. During EMT and initiation of cancer cell invasion, ß-catenin translocates to the nucleus and acts as a transcription factor [41]. Our IF analysis using a ß-catenin-specific antibody demonstrated that expression in mesothelioma was most prominent within the cell body and was minimally present in TnTs (**Figure 3E**). E-cadherin, a 12-kDa transmembrane glycoprotein localized to the adherens junctions of epithelial cells, associates with catenins. Loss of E-cadherin expression is associated with increased invasion, epithelial-to-mesenchymal transition, and tumor progression. TnTs connecting MSTO cells showed inconsistent or minimal expression of this protein (**Figure 3F**). The cytoplasmic linker molecule ZO-1 (zonula occludens) links transmembrane molecules to the actin cytoskeleton in tight junctions [41]. We determined that, in mesothelioma cells forming TnTs, ZO-1 localizes to the cytoplasmic membrane at areas of direct cell-to-cell contact (at the site of gap junctions) and also the site of extrusion of TnTs (**Figures 3G**). These findings indicate that ZO-1 may also be a critical regulator of TnTs.

**Figure 4:**
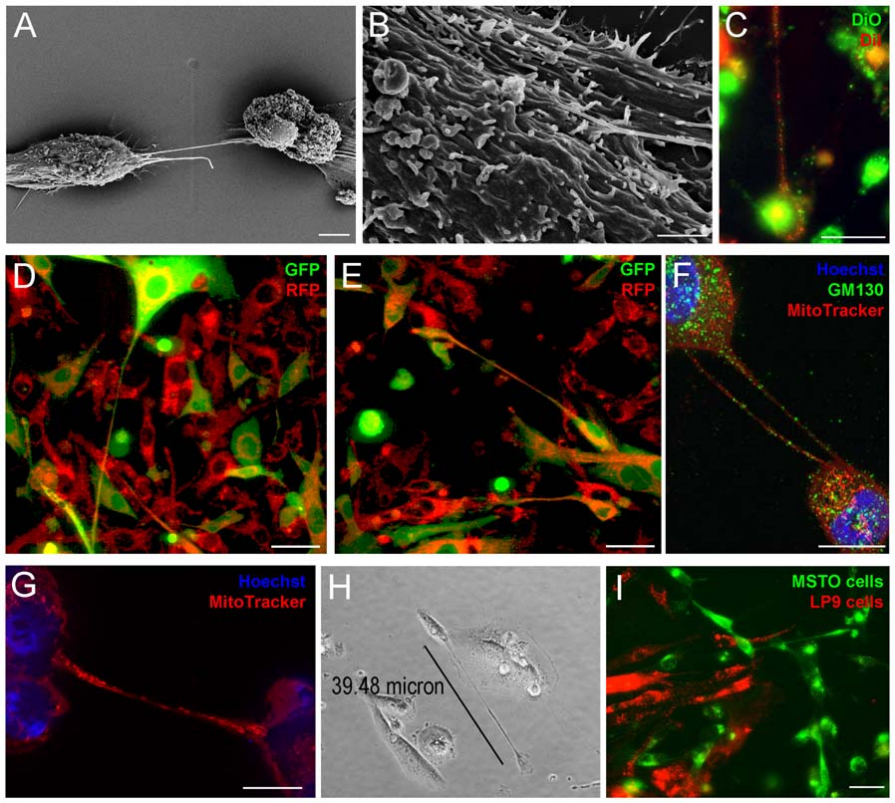
TnTs facilitate intercellular transfer of lipophilic cytosolic components as well as proteins, mitochondria, and golgi vesicles between MSTO-211H cells. a) Electron micrograph of two MSTO-211H cells connected via a TnT. b) Closer view of EM, illustrating that the TnT has more than one insertion point into the membrane of the MSTO cell. c) Cells stained with either green (DiO) or red (DiI) dyes formed TnTs which transmitted lipophilic components when mixed. d) and e) Cells expressing GFP or RFP formed TnTs which readily transmitted these proteins between cells, as demonstrated by time-lapse imaging. f) Fixed cells stained with Hoechst dye, GM130 (for Golgi vesicles), and MitoTracker demonstrated transfer of Golgi and mitochondria as well. g) TnTs relay significant amounts of mitochondria between cells. h) LP9 (normal mesothelial cells) can be induced to form TnTs in a low-serum, hyperglycemic microenvironment. i) Normal mesothelial cells also demonstrate an independent capability of TnT formation among themselves, but do not initiate or form connections to malignant mesothelioma cells via TnTs. LP9 cells stained with DiI (red) were mixed with MSTO-211H cells stained with DiO (green); normal mesothelial cells did not form TnT connections to malignant mesothelioma cells. Scale bars: a) 5 µm, b) 2 µm, c) 30 µm, d) 50 µm, e) 50 µm, f) 10 µm, g) 10 µm, h) 30 µm.

In order to elucidate morphology of TnTs at the cell surface, we performed scanning electron microscopy (EM) of MSTO-211H cells. Interestingly, TnTs appeared to insert below the cell membrane at multiple sites, rather than one single site of entry as initially expected (**Figures 4A and 4B**). The cable-like TnTs inserted into the membrane and extended just under the cell surface for some distance. Scanning EM was utilized to distinguish microvilli from TnTs. Invasive microvilli are slender, branching, actin-based adherent extensions which were demonstrated in a biphasic mesothelioma by Zu and colleagues [42]. The authors' description of invasive microvilli which invaginate into the cytoplasm of neighboring cells differs from TnTs which connect directly with the cytoplasm of the recipient cell. In addition, the presence of microvilli is identified as a characteristic of mesothelioma which distinguishes this cell type from adenocarcinomas; however, we readily noted TnT formation in adenocarcinomas, such as those of lung origin (**Figure 1E**). Our scanning EM images (**Figure 4A**) captured the distinction between the relatively short tentacle-like extensions of microvilli of the mesothelioma cell, in significant contrast to the long, thin, elevated and non-adherent bridge-like TnT which passes over the microvilli and extend to connect distant cells.

### Tunneling nanotubes may form *de novo* and transmit cytosolic components via bidirectional transfer

The timeframe of *de novo* TnT formation was variable. Using 24-hour time-lapse microscopy, we discovered that new TnTs may form over as few as 15 minutes, or over as much as 4 to 5 hours (**Supplemental Movie S4**). Rustom et al. had documented that TnTs may form in as few as 4 minutes between single cells in close proximity [4]. We noted *de novo* formation of TnTs occurring between cells both at close and distant proximity – up to 5 to 6 cell lengths apart – thus likely contributing to disparities in the amount of time needed to form new TnTs. In addition, type II nanotubes – those which form and remain once two connected cells migrate apart – may last even longer than we previously thought, as long as 7 or even 10 hours. We noted evidence of this lengthy time of formation and maintenance of multiple such TnTs in the scratch assay described previously (**Supplemental Movie S4**).

In order to visualize transfer and determine identity of transmitted components, we used fluorophores such as the lipophilic dyes DiI (red fluorescence) and DiO (green fluorescence). Cells were labeled separately with one or the other dye, and the two populations were then co-cultured for up to 24 hours. Some cells in live culture exhibited unidirectional transfer of lipophilic material. In others, we observed evidence of bidirectional transfer, in which red and green lipophilic components intermixed and transferred via TnTs, resulting in a yellowish or lighter color phenotype (**Figure 4C**). To confirm these findings, we transduced cells with either RFP or GFP-expressing lentiviral vectors and repeated the mixing experiments. We noted TnT formation between these two populations when intermixed at variable ratios. GFP and RFP transferred via TnTs in both unidirectional and bidirectional fashion (**Figures 4D and 4E**).

We demonstrated bidirectional transfer of membranous and cytosolic components of mesothelioma cells via TnTs, using lipophilic dyes as well as proteins in the form of RFP and GFP. TnTs have been documented to transmit other cellular agents, such as calcium which induced conductivity in dendritic cells and monocytes [5], [15] as well as in mature macrophages [43], prions between neuronal cells [11], and viruses such as HIV transmission between T cells [44]–[46], B cells [12], and macrophages [47]. Other cell populations noted to develop TnTs to facilitate cell-to-cell transfer include neutrophils [48], normal rat kidney cells (unidirectional transfer) [49] as well as primary human renal epithelium [19], endothelial progenitor cells, which form TnTs to repair cell damage to endothelial cells [50], and cardiomyocytes [51].

In order to assess the ability of mitochondria to be transmitted between mesothelioma cells via TnTs, we used MitoTracker Red to stain MSTO-211H cells which were then cultured in hyperglycemic, low-serum medium. Mitochondria were transmitted between cells in a bidirectional manner (**Figures 4F and 4G**). Thus we concluded that the nanotube-like structures we observed were in fact TnTs capable of transmitting lipophilic cytosolic and membrane components as well as proteins and mitochondria between cells in either a unidirectional or bidirectional manner.

TnTs have been shown to transmit larger cell components such as organelles and endosome-related lysosomes [52]. Bidirectional transfer of mitochondria and intracellular vesicles via TnTs had been previously documented between non-malignant cells such as macrophages, which appeared to form TnTs of greater diameter than we observed in mesothelioma [52], as well as between stromal and renal tubular cells [18] and renal epithelial cells [19]. The importance of mitochondrial transfer and its impact on cancer proliferation remain to be elucidated. Our demonstration of mitochondrial transfer between mesothelioma cells builds upon previous findings that mitochondria may transfer from adult stem or somatic cells with intact functional mitochondria to cells with nonfunctional mitochondria, thus providing rescue of aerobic respiration in these cells [53]. In the setting of the Warburg effect and glycolysis occurring paradoxically under aerobic conditions in cancer cells, the sharing of mitochondria between cancer cells may provide a means for fueling further cancer cell maintenance and proliferation. Also, the demonstration that normal and tumor cells harbor heteroplasmic and homoplasmic mitochondrial DNA mutations – even in a single individual patient with cancer – may implicate TnTs as a method of transfer of genetic change leading to tumor heterogeneity [54].

We also observed nanotube formation in two cell lines of normal (non-malignant) mesothelial LP9 and Met5A cells (**Figure 4H** **and Supplemental Figure S2**). We initially hypothesized that mesothelial cells, when co-cultured with invasive mesothelioma cells, would connect and communicate with cancerous cells via TnTs. We tested this hypothesis by separately adding the fluorophores DiO and DiI to MSTO-211H and to LP9 mesothelial cells, respectively. Interestingly, there appeared to be no evidence of exchange or nanotube formation between mesothelioma and normal mesothelial cells when the populations were mixed (**Figure 4I**). Within the same cultures, there was ample evidence of TnT formation between malignant cells, and also separately between the benign mesothelial cells. We successfully reproduced this finding using several combinations of mesothelioma (MSTO, VAMT) and benign mesothelial (LP9,Met5A) cells (**Supplemental Figure S2**). Upon mixing populations of two mesothelioma cell lines of different histologic type (biphasic MSTO and sarcomatoid VAMT), there was evident nanotube formation between the two different cell lines and mixing of cellular contents (**Supplemental Figure S2D**). These findings suggest that mesothelioma cells produce TnTs that target and attach to cancer-specific transmembrane molecules or receptors that are not found on normal mesothelial cells. Experiments are ongoing to explore this possibility further.

### Suppression of TnT formation

We postulated that suppressing TnT formation would provide further insight into the underlying cellular mechanisms of TnT formation. Prior work had demonstrated that actin-depolymerizing agents such as cytochalasin B and D, Latrunculin A, azide, colchicines, and tubulin inhibitors block TnT formation or traffic along TnTs [19], [46], [52], [55], [56]. In T lymphocytes, toxin B of clostridium difficile and the cdc42-specific inhibitor secramine A also blocked Fas-induced nanotube formation [14].

In order to identify potential metabolic pathways essential for TnT formation, we identified several inhibitors of pathways that have been implicated in actin-based cell invasion. Metabolic inhibitors such as metformin suppress glycolytic migration by inhibiting gluconeogenesis of glioma cells [57]. The mTor pathway has been implicated in actin organization which furthers cancer cell invasion and metastasis [58], [59], and also induces TnT development in astrocytes under conditions of cellular stress [22]. We postulated that an inhibitor of the mTor pathway (Everolimus) would thus interfere with TnT development.

**Figure 5:**
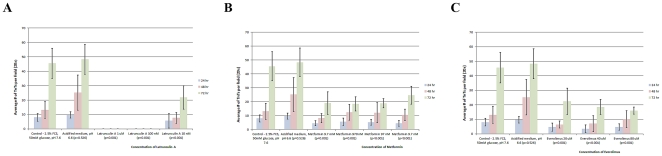
Metabolic compounds suppress formation of TnTs at 0, 24, 48, and 72 hours under low serum, hyperglycemic conditions. Ten visual fields were examined for each medium condition, and the number of nanotubes per field was averaged; each experiment was performed in duplicate (n = 20). a) Effect of Latrunculin A on TnT formation in MSTO cells. b) Effect of Metformin on TnT formation in MSTO cells. c) Effect of mTOR inhibition by Everolimus on TnT formation in MSTO cells.

We examined the impact of metformin, everolimus, and latrunculin A on TnT formation. The compounds were dissolved individually in the low serum/hyperglycemic medium and added to adherent cells. Cells were grown for 3 days in the presence or absence of either compound with the same TnT-inducing medium. The numbers of TnTs were counted in ten high-power microscopic fields every 24 hours, and the results averaged. Each of these compounds significantly suppressed TnT formation by 72 hours of incubation compared with controls. Metformin significantly suppressed TnT formation at all concentrations tested: 9.7 nM, 97 nM, 970 nM, and 9.7 mM (**Figure 5B**). Metformin did not significantly affect cell proliferation (**Supplemental Figure S3**). Everolimus also suppressed TnT formation, at concentrations of 20 µM, 40 µM, and 80 µM (**Figure 5C**). The actin-depolymerizing agent latrunculin A also had pronounced effect on TnTs of mesothelioma cells. At the higher concentrations (1 µM and 100 nM), cells uniformly detached. At the lowest concentration (10 nM), formation of TnTs was significantly decreased (**Figure 5A**).

These results provide evidence that the respective pathways blocked by these compounds either initiate or are key mediators of TnT formation. The suppression of TnTs using these compounds was effective across several mesothelioma cell lines. We are currently exploring the underlying mechanisms of these pathways as they relate to TnT formation and maintenance. As we noted, the hyperglycemia was one component which stimulated increased TnT formation. Under hyperglycemic conditions, the mTor pathway is overactivated. Components of this pathway – in particular, mTorc2 – have been implicated in actin organization [60]. The exact mechanisms and potential implications on TnT formation and resultant intercellular communication remain to be explored. The potential effects of TnT formation on local cell invasion and distant metastasis are being explored in our laboratory.

### The first documentation of TnTs in primary cancer cells and in solid tumors from patients

The process of TnT formation in cancer cells, and the implication of TnT for invasion of malignant cells, had not been explored extensively. The few studies noting observation of TnTs in cancer cells have been limited to cell lines. Rustom et al. first reported tunneling nanotubes in the PC12 cell line (rat pheochromocytoma) [4]. Castro et al. observed TnTs in several cell lines of colon adenocarcinoma [20]. Vidulescu et al. observed movement of vesicles between cells from a human prostate cancer cell line through what they termed ‘intercellular bridges’ which, as described, appear functionally similar to TnTs [6].

We hypothesized that TnTs are present not only in human primary cancer cells cultured *in vitro*, but also in actual solid tumors from patients. We obtained and microsectioned five fresh intact samples of tumors resected from patients with malignant pleural mesothelioma or poorly-differentiated lung adenocarcinoma. The sections were stained with MitoTracker Red and Hoechst 33342 dye. For undetermined reasons, TnTs in tumor tissue sections trapped the blue Hoechst dye, which gave the appearance that DNA material was present and being transported. However, it is possible that the TnTs and/or the tissue specimens as a whole attracted non-specific binding of fluorescent dyes, thus extra care should be taken when immune-staining resected tissues for similar future experiments.

**Figure 6:**
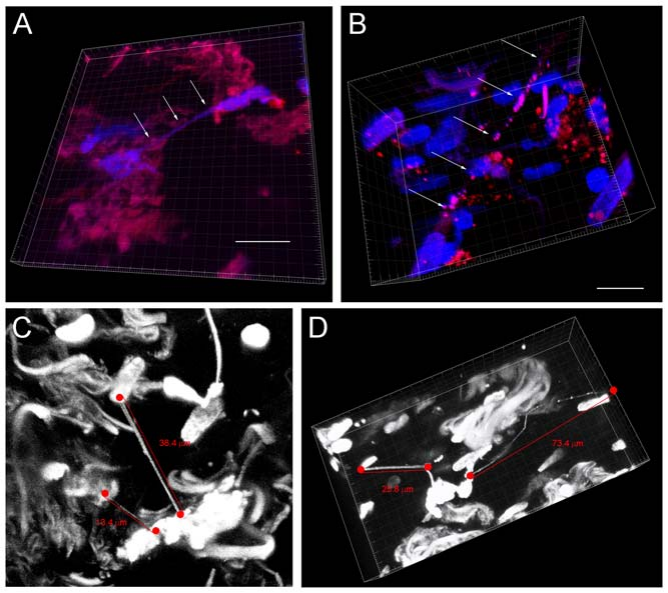
Tunneling nanotubes are present in solid tumors resected from patients with malignant pleural mesothelioma and lung adenocarcinoma. Confocal microscopy was performed and 3-dimensional images constructed using Imaris Viewer. a) Two cells from a mesothelioma solid tumor are connected by a nanotube in three-dimensional plane. b) A long TnT is noted in another mesothelioma tumor specimen. c) Lung adenocarcinoma tumor specimen also manifesting multiple intact nanotubes of various lengths. d) Nanotubes present in a lung adenocarcinoma tumor sample vary in length, width, and extent of curvature. Multiple “blebs” are noted in the lengthy, curved nanotube on the right, measuring at least >70 µm. Scale bars: a) 15 µm, b) 15 µm; c) and d) as noted in the figure.

3-dimensional reconstruction of these images revealed that TnTs were present in all five of the tumor specimens we had obtained from patients (**Figure 6A** **and Supplemental Movie S5**). These nanotubes were noted to be long, thin, and suspended in the stroma of the tumor matrix (**Figure 6B–D**). Some TnTs were straight and others curved in a similar manner to the dendritic cell corneal model noted by Chinnery et al. [61]. Microvesicle blebs were readily visible along the nanotubes as well, identical in appearance to what we noted in TnTs *in vitro*.

To date, there has been only one published study demonstrating potential *in vivo* presence of nanotubes. Chinnery et al. successfully imaged dendritic cells forming long, thin, curved nanotubes in a mouse cornea model by inducing inflammation [61]. Our successful demonstration of TnTs in several solid tumor samples opens the door to pursuing examination of TnTs in *in vivo* subjects and assessing intercellular communication processes in progress.

### Conclusions and future directions

To our knowledge, we provide the first evidence of TnTs in mesothelioma and also the first demonstration of TnTs in primary cancer cells and intact tumors from patients with mesothelioma and lung adenocarcinoma. We discovered that TnT formation is most effectively stimulated and accelerated in a hyperglycemic, low-serum, acidic environment. IF staining of fixed cells with TnTs, and time-lapse imaging of live stained cells demonstrated both unidirectional and also simultaneous bidirectional transfer of proteins, mitochondria, Golgi, and other cytosolic components. We also demonstrated the ability of chemical suppression of TnT formation using inhibitors of various metabolic signaling pathways, such as metformin, everolimus, and latrunculin A.

The potential roles of TnTs in cancers *in vivo* are numerous. For example, it is possible that they may propagate chemotherapy resistance via intercellular transfer of proteins responsible for causing and/or maintain resistance [62]; the recent discovery of antibiotic resistance of bacteria following communication via TnTs [63] provides further support for the idea that cancer cells may also be capable of acquiring genes which induce resistance to chemotherapy in this fashion as well. Having demonstrated the presence of TnTs in human cancer and their ability to propagate intercellular communication, we believe that these findings support the idea that this process plays an important role in cancer cell biology and warrants further evaluation. We propose that TnTs are critical to providing a direct cytoplasmic connection between invasive malignant mesothelioma cells and play an important role in cancer cell pathogenesis and invasion.

## Materials and Methods

### Cell Lines and Culture Medium

MSTO-211H, VAMT, and H-Meso cell lines were obtained courtesy of Dr. Yuman Fong, MSKCC. MSTO-211H is derived from a patient with biphasic mesothelioma (ATCC no. CRL-2081). VAMT is a sarcomatoid mesothelioma cell line. H-Meso is an epithelioid mesothelioma cell line. All cell lines were passaged in plasmocin-containing medium (Invivogen, San Diego, CA) and tested negative for mycoplasma contamination Cell lines were authenticated by the Core Fragment Analysis Facility at Johns Hopkins University using short tandem repeat profiling in August 2010. We also used two human mesothelial (benign) cell lines: LP9 (LP9/TERT-1) is an hTERT-immortalized cell line derived from human peritoneal mesothelium [64], [65]; Met5A is an immortalized human mesothelial cell line (ATCC CRL-9444) derived from the pleural lining [66]. LP9 cells were grown in 15% M199 medium with 10 ng/ml EGF (Peprotech, Invitrogen), 50 ng/ml hydrocortisone (Sigma), 1% penicillin-streptomycin, 2% glutamine. Met5A cells were cultured in 10% FCS in M199/MCDB105 (1∶1) with 100 U/ml penicillin-streptomycin and 2% L-glutamine. All cell lines and primary cells were passaged every 2–3 days using 0.25% (w/v) Trypsin-0.53 mM EDTA solution.

Mesothelioma cells were passaged using 10% fetal calf serum (FCS) in RPMI-1640 with 25 mM glucose, supplemented with 1% penicillin-streptomycin (P-S) and 2% L-glutamine, at normal pH (7.6). To stimulate nanotube formation, cells were grown in 2.5% FCS in RPMI-1640 containing 50 mM glucose, supplemented with 1% P-S, 2% L-glutamine, and 10 mM ammonium lactate [67] (Sigma Aldrich, St. Louis, Missouri); this medium was acidified to pH 6.6. To induce EMT, the following reagents were used: 10 ng/ml porcine pancreas-derived insulin (Sigma); 100 ng/ml cholera toxin (Biosciences VWR International); 0.5 µg/ml hydrocortisone (Sigma), 20 ng/ml EGF (PeproTech, Invitrogen), 2 ng/ml FGF (Peprotech, Invitrogen), and TGF-ß3 (Invitrogen). All cultures were grown in 75 cm^2^ tissue culture flasks (Falcon, Becton Dickson, Oxnard, CA) at 37°C in 5% CO_2_.

### Human Tumor Cells and Samples

Histologically-confirmed mesothelioma and lung adenocarcinoma cells from patients with pleural effusions were obtained via an MSKCC Institutional Review Board (IRB)-approved protocol. Informed written consent was obtained from all patients, and patient identifiers were removed to ensure anonymity. In addition, fresh intact tumor specimens from five patients with malignant pleural mesothelioma or poorly-differentiated lung adenocarcinoma were obtained, and placed in phosphate-buffered saline (PBS). Vibratome sections (100–300 µm thick) were cut and stained using Hoechst 33342 (10 µg/ml) and MitoTracker Red dyes (500 nM). The stained sections were mounted between two glass coverslips and imaged on a confocal microscope (see below).

### Viral Transfection of Cell Lines

GFP and RFP-expressing MSTO-211H cells were generated using lentiviral vectors expressing each protein. To generate the respective virus, 293T cells were transfected with the lentiviral vector pUltra (Addgene plasmid 24129) encoding GFP or pUMW-PTB encoding btomato/RFP (Dogan et al., manuscript in submission). The viral supernatants were overlaid over semi-confluent MSTO-211H cells in culture flasks for one hour before 10% FCS RPMI-1640 medium was added. Cultures were incubated for 48 hours before imaging.

### Viable Staining of Cell Lines

The cells were cultured in clear-bottomed delta-T culture dishes (Bioptechs Inc., Butler, PA). The fluorescent lipophilic dyes DiI and DiO (Multicolor Cell-Labeling kit, Invitrogen, Molecular Probes) were used per manufacturer's instructions. In a separate set of experiments, MitoTracker Red CMX Ros (Invitrogen) was used at 500 nM to stain mitochondria, per manufacturer's protocols. Stained cells were re-suspended and added to a non-confluent culture of adherent, unstained MSTO-211H cells grown in another dish. Incubation was performed in high glucose medium for five hours to stimulate formation of TnTs prior to imaging.

### Glucose Stimulation of TnT Formation

To determine average numbers of nanotubes under various growth conditions, MSTO-211H cells were cultured in 6-well adherent tissue culture plates (Fisher Scientific, Pittsburgh, PA) using RPMI-1640 medium and 2.5% FCS at normal pH 7.6 with 25 mM or 50 mM glucose; RPMI-1640 medium with 10% FCS with 25 mM or 50 mM glucose; or the same media used at low pH (6.6). The number of nanotubes was counted in 10 fields per medium condition, at regular time intervals (0, 24, 48, and 72 hours) using a 20× objective lens on a Nikon Eclipse Ti inverted microscope (Nikon Instruments, Inc.) and the results averaged. Experiments were performed in duplicate.

### Assessment of Cell Proliferation

MSTO cells were cultured as described above for the experiments to assess number of nanotubes forming over time. Cells were visualized using confocal microscopy and also counted in five separate 20× fields per medium condition and per timepoint; the experiments were performed in duplicate, for a total of ten fields counted for each condition and timepoint. The results were averaged and graphed using Microsoft Excel. Standard errors were calculated and depicted as shown.

### Pharmacological Treatment of Cell Lines

Metformin (Sigma), Latrunculin A (BioMol International, and Everolimus (Afinitor™, Novartis) were used in variable concentrations as stated in the Results. The number of non-adherent nanotubes was counted in 10 fields per medium condition, at regular time intervals (0, 24, 48, and 72 hours) using a 20× objective lens on a Nikon Eclipse Ti inverted microscope (Nikon Instruments, Inc.) and the results averaged. Experiments were performed in duplicate.

### Fixation and Sample Preparation

To prepare cells for IF staining, cells were cultured in one- or two-well sterile tissue culture-treated chamber slides (Lab-Tek II Chamber Slide™ system, Nunc, Rochester, NY) or on sterile poly-L-lysine (1 mg/ml; Sigma) coated glass coverslips for 48–72 hours.

To perform fixation and prevent disruption of existing nanotubes, 16% w/v paraformaldehyde (PFA) (Alfa Aesar, Ward Hill, MA) was added along the sides of the chambers or the dishes with glass coverslips, keeping the overlying culture medium intact to a final w/v concentration of 4%, After incubation at 4°C for 1–2 hours, the fixative and chambers were removed, and slides were allowed to air dry. Fluorescent staining was performed to detect for presence of various proteins as follows.

### Phalloidin staining of cell cultures

Fluorescent staining for actin was performed with phalloidin-rhodamine (Molecular Probes) used at 4 units/ml in culture medium with 0.05% Triton X. The stain was added to live cells with incubation at 37°C for 15 minutes prior to fixation

### Immunofluorescent Staining

The primary antibodies and their working concentrations are as follows: rabbit anti-β-catenin (Sigma, 5 µg/mL), mouse anti-Ezrin (Santa Cruz, 5 µg/mL), mouse anti-E-Cadherin (BD Bioscience, 2.5 µg/mL), mouse anti-human Fascin (Dako, 1 µg/mL), mouse anti-ZO1 (Zymed, 1 µg/mL), mouse anti-GM130 (BD Transduction, 1 µg/mL). Slides were first blocked with blocking solution (10% normal goat serum/2% BSA in PBS) or mouse IgG blocking agent from Vector Labs for 30 min. Primary antibody incubation lasted 3 to 7 hours at room temperature, followed by 30 minutes incubation with biotinylated secondary antibodies (Vector Labs, 1∶200 dilution) incubation. Detection was performed with Streptavidin-HRP D (Ventana Medical Systems) followed by Tyramide-Alexa Fluor 488 (Invitrogen).

### Optical Imaging of Fixed Samples

Confocal imaging of fixed samples were performed using Zeiss LSM 5Live line-scanning or Leica SP2 point-scanning microscopes using Zeiss oil 40×/1.3NA Plan-Neoflur, Zeiss oil 63×/1.4NA Plan-Apochromat or Leica water 63×/1.2NA HCX PL APO CS objectives. Serial z-stack images were obtained at optimal step size and maximum intensity projection images were produced.

### Time-lapse Imaging of Live Samples

Time-lapse imaging experiments were performed on Perkin Elmer UltraView ERS spinning-disk confocal microscope or Zeiss LSM 5Live line-scanning confocal microscope. Both microscopes were enclosed in environmental chambers that were maintained at 37°C with 5% CO_2_ level.

For determining timeframe of TnT formation, brightfield time-lapse images were taken every 15 minutes for 24 hours. For all other time-lapse experiments, DIC (differential interference contrast) or phase contrast images were taken in addition to fluorescent imaging. Acquisition frequency was every 3-4 minutes for up to 3 hours.

### Electron-Microscopic Imaging of Nanotubes

To perform scanning and transmission EM, 1–3×10^6^ MSTO-211H cells were cultured on Thermanox plastic tissue culture 25 mm cover slips (Lux Scientific Corporation). The fixative −2.5% glutaraldehyde/2% paraformaldehyde in 0.075 M sodium cacodylate buffer (pH 7.5; 10 ml, Electron Microscopy Sciences, Hatfield, PA) – was added directly to the overlying medium.

### Image Processing

The Imaris Viewer program (Bitplane Scientific Software, Inc.) was used to construct and visualize 3-dimensional images of the tumor samples. Metamorph (Molecular Devices) image analysis software was used to process time-lapse data and to create still images and movies.

### Statistical Analysis

For analyses in Figures 2 and 5, the two-sided chi-square test was used to determine significance. P values less than 0.05 were considered significant. For Figure 2, each medium condition was considered an independent experiment therefore correction for multiple comparisons was not performed. For Figure 5, each drug treatment was considered an independent experiment therefore correction for multiple comparisons was not performed. For Figures S1 and S3, standard errors were calculated in Microsoft Excel and graphed as shown.

## Supporting Information

Figure S1 **Proliferation curves of MSTO cells cultured under variable medium conditions (variable serum (10% or 2.5% FCS), glucose concentration (25 mM or 50 mM), and pH (7.6 or 6.6)).** Cells were counted at 24, 48, and 72 hours in a 20× field, and average values were graphed as shown. A) MSTO cells cultured in four separate medium conditions at normal pH (7.6). B) MSTO cells cultured separately in the same media acidified to pH 6.6. Among cells cultured in medium of normal pH (7.6): proliferation rate of cells is essentially the same for the first 48 hours; by 72 hours, there is a small variation. At 72 hours, the lowest cell count is for the low serum/hyperglycemic medium (the condition which stimulates higher nanotube formation), and the highest cell count is for the high serum (10% FCS), normal glucose (i.e. common medium conditions for cell passaging), as expected. Among cells grown in 10% FCS: cells in hyperglycemic medium had less proliferation. Cells grown in low serum (2.5% FCS) demonstrated less proliferation in hyperglycemic medium. Overall, proliferation was more prolific in cells cultured in 10% FCS, not in low serum conditions. Among cells cultured in acidic medium (pH 6.6): cell growth was similar among conditions over the first 24 hours; however by 72 hours, a higher proliferation rate was noted using 10%FCS, while TnT formation was more prominent in the 2.5% FCS group. Proliferation was lowest in cells grown in 2.5% FCS, as expected, and with low glucose. Highest rate of growth was in high serum/hyperglycemic conditions. Among all conditions, highest cell proliferation occurred with 10% FCS, normal glucose, and normal pH. Lowest proliferation rate was among cells grown in 2.5% FCS, normal glucose, and acidic pH. In all cases, cells grown in acidic pH had a lower growth curve than cells grown at normal pH. It was notable that cells grown in 10% FCS/25 mM glucose (passage medium) had double the growth rate compared to cells grown in low serum 2.5% FCS with high glucose (i.e. the medium we used to stimulate nanotube formation). This finding of decreased proliferation of cells in hyperglycemic medium is consistent with previously published studies [23].
(TIF)

Figure S2 **Normal mesothelial cells are also independently capable of TnT formation, but do not appear to form TnTs with malignant mesothelioma cells. However, malignant mesothelioma cells of different histopathology (biphasic and sarcomatoid) do form TnTs with each other and exchange cytosolic materials.** Multiple cell lines of normal mesothelium (LP9, Met5A) and malignant mesothelioma (MSTO, VAMT) were stained with lipophilic dyes DiI (red) or DiO (green), or MitoTracker Red; in panels a) and b), MSTO cells expressing GFP were used. Cells were cultured for 48–72 hours and examined for formation of nanotubes. Mixing of LP9 with MSTO occurred without TnT formation between the two populations, and is demonstrated in Figure 4I. a) GFP-expressing MSTO cells (green) were mixed in 1∶1 ratio with Met5A mesothelial cells stained with DiI (red). Nanotube formation was observed between cells of the same cell type, but not between different cell types. b) Another demonstration of TnT formation between like cells, but not between MSTO (green) cells and Met5A (red) cells. c) VAMT (sarcomatoid) mesothelioma cells were stained with MitoTracker Red (indicated by arrowheads), and co-cultured with unstained Met5A cells (noted by asterisk, *) to examine potential transfer to normal mesothelial cells. There was no evidence of such transfer. d) VAMT (DiI, red) and MSTO (DiO, green) cells were co-cultured for 48 hours. TnT formation occurred (arrows). Yellow fluorescence resulted from mixing of red and green dye, an was thus indicative of intercellular exchange between these two histologic subtypes of mesothelioma.
(TIF)

Figure S3 **Assessment of potential effects of metformin on cell proliferation. MSTO cells were cultured for 72 hours in either usual passage medium (10% FCS, 25 mM glucose RPMI) or 2.5% FCS, 50 mM glucose RPMI (control medium), with or without metformin (final concentration 100 nM or 1000 nM). Average numbers of cells and TnTs per 20× field were graphed as shown. Metformin caused essentially no effect on cell proliferation under either medium condition, but effectively suppressed TnT formation in the low-serum, high-glucose environment.** A) Proliferation of MSTO cells in 10% FCS, 25 mM glucose RPMI medium (control), with or without metformin. B) Average number of TnTs in cells cultured in 10% FCS, 25 mM glucose RPMI. C) Proliferation of MSTO cells in 2.5% FCS, 50 mM glucose RPMI medium (control), with or without metformin. D) Average number of TnTs in cells cultured in 2.5% FCS, 50 mM glucose RPMI.
(TIF)

Movie S1 **Real-time movie of MSTO cells in culture with intact TnTs.** Cells were cultured in 6-well tissue culture plates to partial confluence for 48 hours in RPMI medium with 2.5% FCS, 50 mM glucose. TnTs formed long “bridges” connecting cells at considerable distance apart. The non-adherence and oscillation of the long TnTs was readily apparent upon movement of the plate.
(MPG)

Movie S2 **An additional real-time movie of MSTO cells in culture with intact TnTs.** Media conditions were the same as described for Movie S1.
(MPG)

Movie S3 **(Time-lapse). Migrating mesothelioma cells demonstrate significant formation of TnTs.** A scratch assay was performed by growing MSTO-211H cells for 72 hours in low-serum, hyperglycemic, acidic RPMI-1640. A micropipette tip was used to create a gap, and the medium was replaced with fresh medium. Time-lapse brightfield confocal microscopy was performed every 30 minutes for 24 hours, demonstrating formation of multiple TnTs along the advancing cell front. White arrows were inserted and appear at 11.5 hours and 14 hours to highlight TnTs. The white arrowhead appearing at 16 hours tracks apparent movement of cargo in a TnT.
(AVI)

Movie S4 **(Time-lapse). Tunneling nanotubes may form by extension of cells at some distance apart, and form a connection to these cells leading to visible unidirectional transmission of cellular cargo.** Brightfield time-lapse imaging was performed every 15 minutes over a 24-hour period. Following an initial thick filopodial cell extension which quickly retracts, three TnTs readily extend in a directional manner toward another group of cells. Upon extension and attachment, multiple cargo blebs can be seen traveling via the TnT from the initiating cell toward the receiving cell.
(AVI)

Movie S5 **3-dimensional reconstruction of the human tumor depicted in** **Figures 6C and 6D** **.** This tumor was resected from a patient with lung adenocarcinoma. 3-dimensional imaging was performed using the Imaris Viewer. Two nanotubes are evident in the longitudinal axis. The longer of the two nanotubes is greater than 70 µm in overall length and encompasses small blebs consistent with vesicular cargo.
(AVI)
