# Supplementary figures and images for: Tunneling Nanotubes Provide a Unique Conduit for Intercellular Transfer of Cellular Contents in Human Malignant Pleural Mesothelioma
Source: PLoS One. 2012 Mar 9;7(3):e33093. doi: 10.1371/journal.pone.0033093 (PMC3302868; doi:10.1371/journal.pone.0033093)

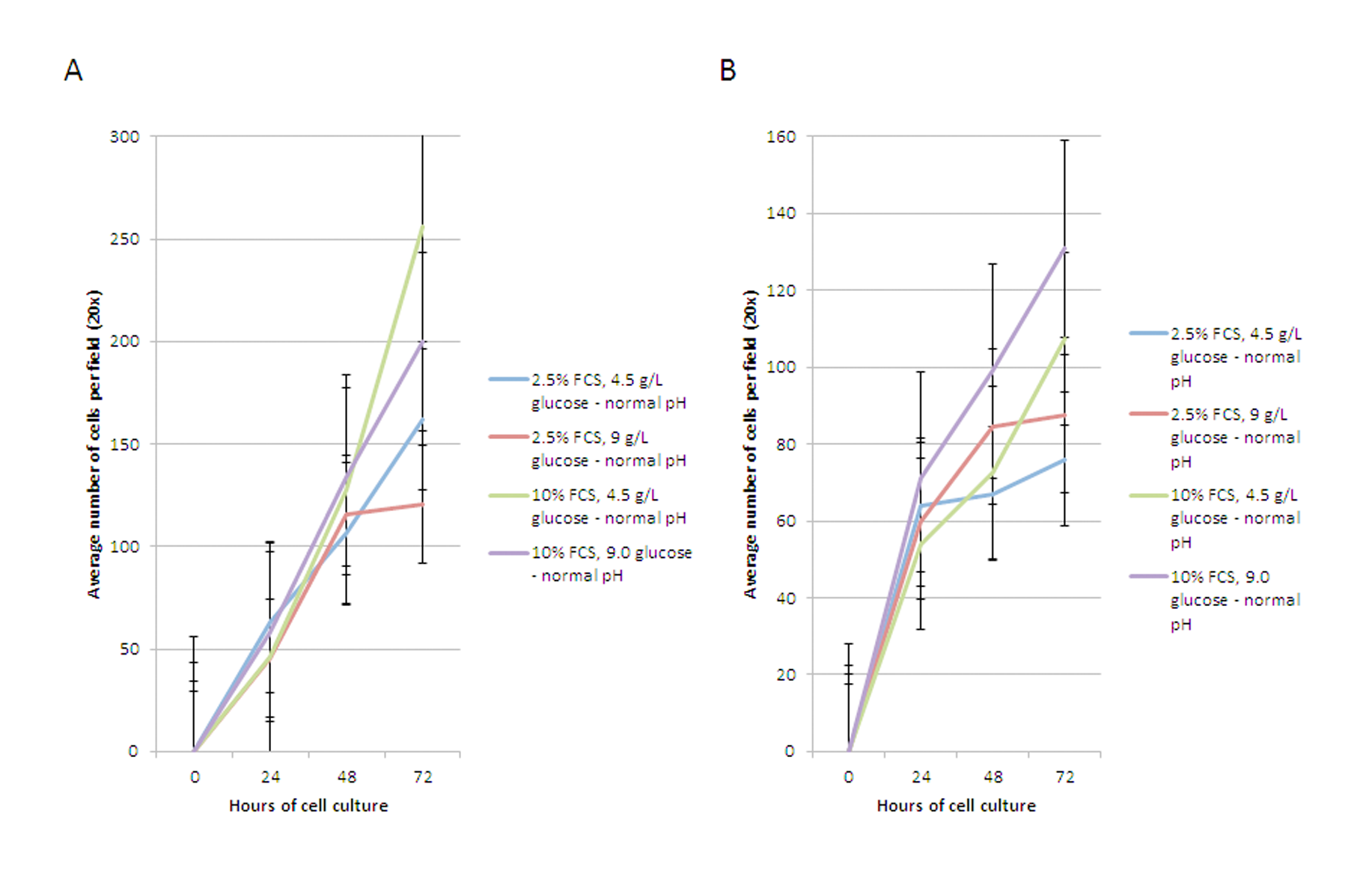

Supplement: Figure S1 — Proliferation curves of MSTO cells cultured under variable medium conditions (variable serum (10% or 2.5% FCS), glucose concentration (25 mM or 50 mM), and pH (7.6 or 6.6)). Cells were counted at 24, 48, and 72 hours in a 20× field, and average values were graphed as shown. A) MSTO cells cultured in four separate medium conditions at normal pH (7.6). B) MSTO cells cultured separately in the same media acidified to pH 6.6. Among cells cultured in medium of normal pH (7.6): proliferation rate of cells is essentially the same for the first 48 hours; by 72 hours, there is a small variation. At 72 hours, the lowest cell count is for the low serum/hyperglycemic medium (the condition which stimulates higher nanotube formation), and the highest cell count is for the high serum (10% FCS), normal glucose (i.e. common medium conditions for cell passaging), as expected. Among cells grown in 10% FCS: cells in hyperglycemic medium had less proliferation. Cells grown in low serum (2.5% FCS) demonstrated less proliferation in hyperglycemic medium. Overall, proliferation was more prolific in cells cultured in 10% FCS, not in low serum conditions. Among cells cultured in acidic medium (pH 6.6): cell growth was similar among conditions over the first 24 hours; however by 72 hours, a higher proliferation rate was noted using 10%FCS, while TnT formation was more prominent in the 2.5% FCS group. Proliferation was lowest in cells grown in 2.5% FCS, as expected, and with low glucose. Highest rate of growth was in high serum/hyperglycemic conditions. Among all conditions, highest cell proliferation occurred with 10% FCS, normal glucose, and normal pH. Lowest proliferation rate was among cells grown in 2.5% FCS, normal glucose, and acidic pH. In all cases, cells grown in acidic pH had a lower growth curve than cells grown at normal pH. It was notable that cells grown in 10% FCS/25 mM glucose (passage medium) had double the growth rate compared to cells grown in low serum 2.5% FCS with high g [file pone.0033093.s001.tif]

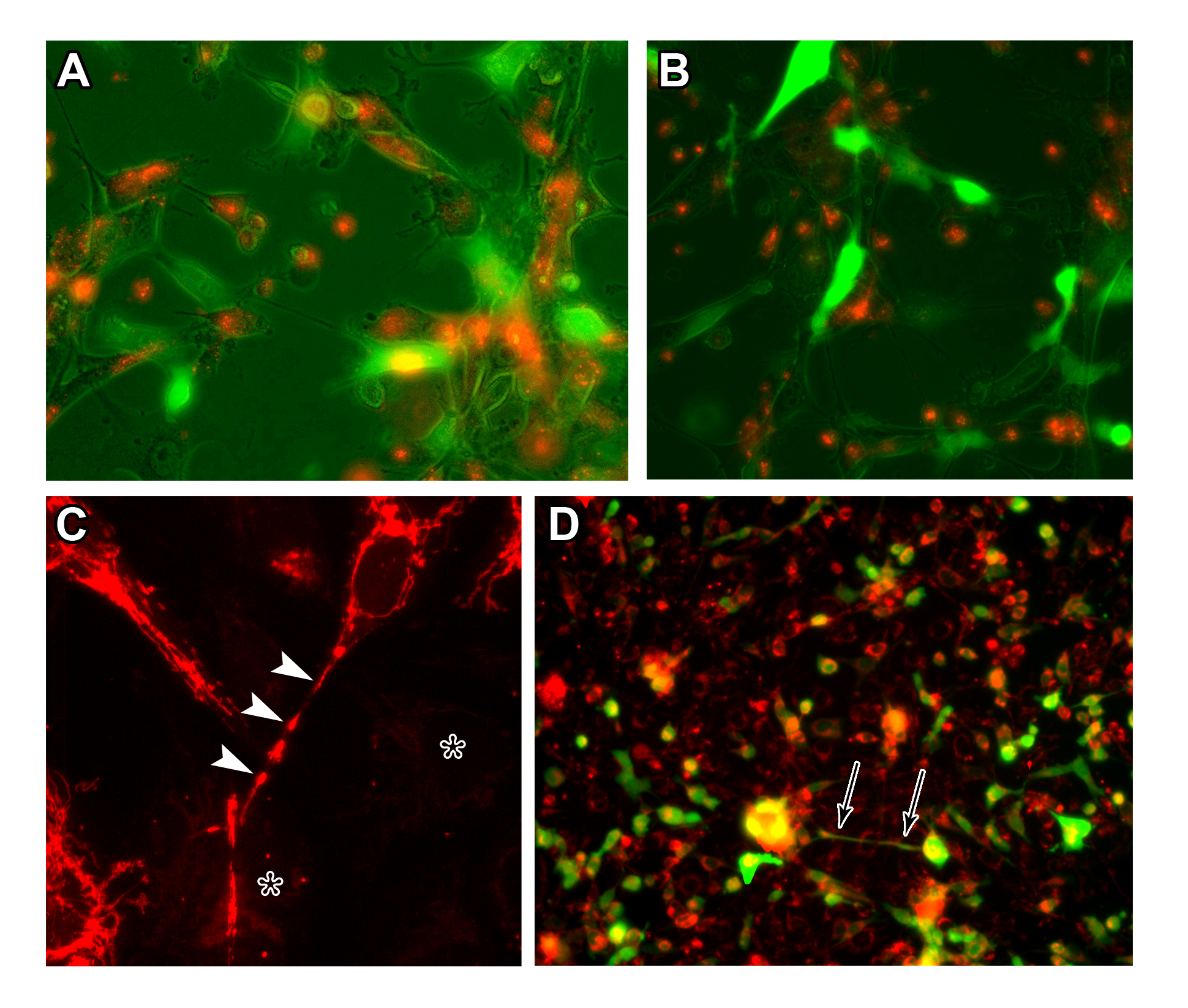

Supplement: Figure S2 — Normal mesothelial cells are also independently capable of TnT formation, but do not appear to form TnTs with malignant mesothelioma cells. However, malignant mesothelioma cells of different histopathology (biphasic and sarcomatoid) do form TnTs with each other and exchange cytosolic materials. Multiple cell lines of normal mesothelium (LP9, Met5A) and malignant mesothelioma (MSTO, VAMT) were stained with lipophilic dyes DiI (red) or DiO (green), or MitoTracker Red; in panels a) and b), MSTO cells expressing GFP were used. Cells were cultured for 48–72 hours and examined for formation of nanotubes. Mixing of LP9 with MSTO occurred without TnT formation between the two populations, and is demonstrated in Figure 4I. a) GFP-expressing MSTO cells (green) were mixed in 1∶1 ratio with Met5A mesothelial cells stained with DiI (red). Nanotube formation was observed between cells of the same cell type, but not between different cell types. b) Another demonstration of TnT formation between like cells, but not between MSTO (green) cells and Met5A (red) cells. c) VAMT (sarcomatoid) mesothelioma cells were stained with MitoTracker Red (indicated by arrowheads), and co-cultured with unstained Met5A cells (noted by asterisk, *) to examine potential transfer to normal mesothelial cells. There was no evidence of such transfer. d) VAMT (DiI, red) and MSTO (DiO, green) cells were co-cultured for 48 hours. TnT formation occurred (arrows). Yellow fluorescence resulted from mixing of red and green dye, an was thus indicative of intercellular exchange between these two histologic subtypes of mesothelioma. (TIF) [file pone.0033093.s002.tif]

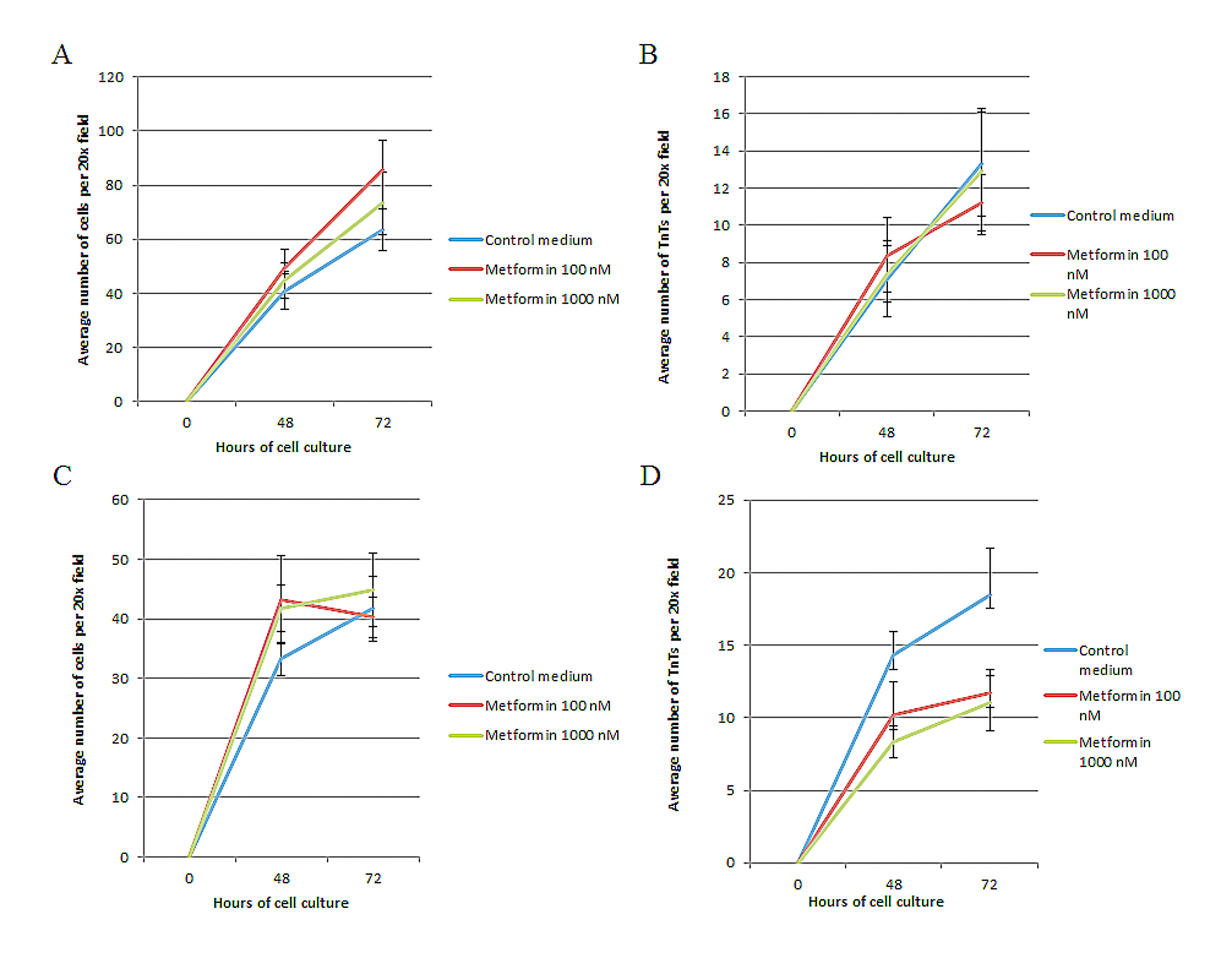

Supplement: Figure S3 — Assessment of potential effects of metformin on cell proliferation. MSTO cells were cultured for 72 hours in either usual passage medium (10% FCS, 25 mM glucose RPMI) or 2.5% FCS, 50 mM glucose RPMI (control medium), with or without metformin (final concentration 100 nM or 1000 nM). Average numbers of cells and TnTs per 20× field were graphed as shown. Metformin caused essentially no effect on cell proliferation under either medium condition, but effectively suppressed TnT formation in the low-serum, high-glucose environment. A) Proliferation of MSTO cells in 10% FCS, 25 mM glucose RPMI medium (control), with or without metformin. B) Average number of TnTs in cells cultured in 10% FCS, 25 mM glucose RPMI. C) Proliferation of MSTO cells in 2.5% FCS, 50 mM glucose RPMI medium (control), with or without metformin. D) Average number of TnTs in cells cultured in 2.5% FCS, 50 mM glucose RPMI. (TIF) [file pone.0033093.s003.tif]
